# Can Green Credit Policies Accelerate the Realization of the Dual Carbon Goal in China? Examination Based on an Endogenous Financial CGE Model

**DOI:** 10.3390/ijerph20054508

**Published:** 2023-03-03

**Authors:** Qianyi Du, Haoran Pan, Shuang Liang, Xiaoxue Liu

**Affiliations:** 1 School of Economics and Resource Management, Beijing Normal University, Beijing 100875, China; 2 Center for Innovation and Development Studies, Beijing Normal University, Zhuhai 519087, China; 3 School of Economics, Beijing Technology and Business University, Beijing 100048, China

**Keywords:** green credit, green technology innovation, computable general equilibrium analysis, dual carbon goals, economic effect, environmental effect

## Abstract

Green credit is an indispensable funding source through which China can achieve its carbon neutrality goal. This paper quantifies the influences of different green credit scales on energy structures, carbon reduction, the industrial economy, and the macroeconomy. It creates a green credit mechanism related to green technology innovation in a Chinese carbon neutrality computable general equilibrium (CGE) model and integrates energy, environmental, economic, and financial (3EF) systems. The green credit scale can influence green technology innovation and hence CO_2_ emissions. The results show that (1) green credit can accelerate China’s achievement of its carbon neutrality goal, and the larger the green credit scale, the less time it takes to achieve goals; (2) the influence of green credit scales confers marginal decreasing effects with realistic policy considerations; (3) using a cost–benefit perspective, 60% is the most appropriate green credit scale to use to achieve dual carbon goals in China; (4) the different green credit scales have a heterogeneous impact on the industry output, and high-carbon-emission producers from nonenergy industries need to pay attention to their green credit risk. This research provides a scientific reference for the policy design of China’s future green financial market development.

## 1. Introduction

In recent years, driven by the growing concern about climate change, there has been a significant increase in the number of international academic studies conducted on green finance. Green finance, which is also referred to as “climate mitigation finance” [1] or “sustainable finance” in research [2], is closely related to carbon peak and carbon neutrality goals.

Green finance refers to investment and financial activities that generate environmental benefits that support sustainable development, including the reduction in air, water, and soil pollution; the lowering of greenhouse gas emissions; the improvement of resource efficiency; climate mitigation and adaptation; and the reflection of their synergistic effects [3]. The international community’s understanding of green finance is divided into two main categories: one is to consider green finance as an investment and financial activity that can improve the environment, i.e., green investment [4,5]; the other focuses on the incorporation of environmental factors into financial investment [6]. The definition of “green finance” in this paper mainly refers to the former. China first gave an official definition of green finance in “Guiding Opinions on Building a Green Financial System” in 2016, where green finance refers to economic activities that support environmental improvement, climate change, and the economical and efficient use of resources.

In 2016, countries were estimated to require USD 5–7 trillion per year to reach the SDGs, and it was projected that developing countries will face an annual investment gap of USD 2.5 trillion [7]. In 2021, more than USD 1.6 trillion in sustainable debt instruments was issued, which brought the total market share to more than USD 4 trillion. Bloomberg showed that green bonds (USD 620 billion in issuance) and sustainability-linked loans (USD 400 billion) were undergoing rapid development while the growth of green loans (USD 100 billion) remained stable in 2021 [8]. In emerging economies, the sustainable debt issuance in 2021 surged to almost USD 200 billion, wherein China was the second largest issuer globally [9]. The maturity of green finance products and services varies between developed and developing countries. Bank systems play an essential role in the financial systems of developing countries. Therefore, green credit is a pre-eminent part of the green financial system in developing countries.

The concept of green credit incorporates sustainable lending, equatorial principles, and corporate social responsibility [10]. The banking industry began to focus on the legal risks associated with the environmental performance of bank loans or credit recipients in 1980 with the enactment of the US Comprehensive Environmental Response Compensation and Liability Act (CERCLA), which spurred international organizations to advocate for sustainable development policies and guidelines across all banks [11]. Increasingly, central banks are being tasked with the implementation of policies that support sustainable growth, mitigate climate finance risks, and achieve national climate change policy objectives [12].

**Figure 1:**
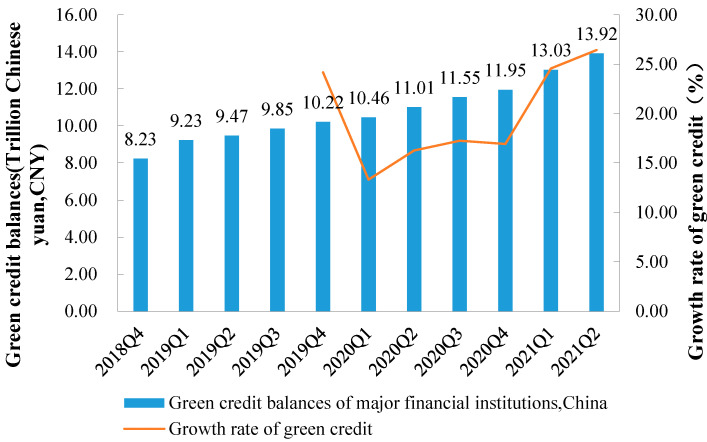
Size and growth rate of green credit in China, 2018–2021. Note: Data are from China Development Bank.

**Figure 2:**
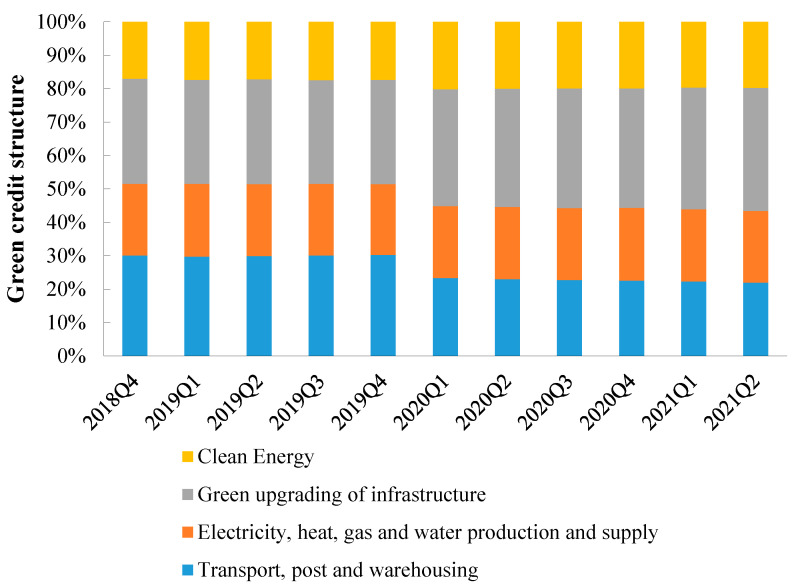
Sector structure of green credit in China, 2018–2021. Note: Data are from China Development Bank.

In China, the current practice of green finance in commercial banks is mainly based on traditional green credit [10,13]. The overall level of green credit development in China is not high [14,15], but the year-on-year growth rate of green credit has been between 10% and 14% in recent years, showing a stable growth trend (Figure 1). From an industry perspective, the leading industries in the investment of green credit in China are the clean energy industry; the electricity, heat, gas, and water production and supply industry; the transportation, storage, and postal industry; and the green infrastructure industry. The proportion of green credit used by nonenergy sectors is as high as 80% (Figure 2).

China has been providing green credit as an economic instrument to enter the financial market since 2007 by way of a policy regime, and over the past fifteen years, the country has gradually formed the current green credit system [16,17]. In recent years, China has been focusing more intensively on the role of green credit as a means of encouraging sustainable development over the next 40 years [18,19]. In particular, in 2021, the China Daily website reported that the aim of the China Development Bank is for green loans to account for about 30% of credit assets by 2030. On this basis, China will rapidly promote green credit and prioritize it over traditional brown credit in the future. China will accelerate the elimination of investment in traditional industries and guide investment flow toward green areas in the form of a rigid policy system. Thus, under policy regulations, the proportion of green credit may reach 60% or 90% in the future. China’s green credit policy will have significant impacts on energy structures, CO_2_ emissions, the industrial and macroeconomy [20,21]. Therefore, this study has forward-facing policy significance as it considers the green credit scale set in China’s green credit policy as an external shock in the economic system and quantifies its impact on the achievement of the carbon neutrality goal.

Recent studies have shown that China can achieve the “2030 carbon peak” goals through carbon-pricing policies [22,23]. However, it will be difficult to achieve the long-term carbon-neutral goal by relying only on carbon taxes and carbon-trading-system policies [24]. Additionally, there is a consensus in the research field that achieving this goal will depend more heavily on green technology innovation [25,26,27]. Green technology innovation can promote coal consumption substitution and energy transformation as well as the upgrading of, and an increase in, the production proportion of clean energy sources, such as solar and wind energy [28,29], but this will require a large amount of green investment to stimulate green technology innovation [30]. Therefore, green credit, the main green financing instrument in China, is required to provide real financial support during the process of green innovation in China.

The purpose of this paper is to test the role of green credit development in China at both the economic and environmental levels and to provide scientific advice to government departments on green credit policy formulation within the constraints of the dual carbon goal. In fact, there is a lack of historical policy experience for developing countries that need to achieve green economies. Because each country has different development modes and pillar industries as well as various industrial linkages, it is impossible to refer to the policy formulations of countries that have already achieved “peak carbon” status or are on the verge of achieving a “carbon neutral” status. Thus, this model may act as an important tool for these countries. Furthermore, this model is replicable. It can be used in different countries if the SAM table can be reconstructed to fit the circumstances of other countries (See Supplementary Materials). The model can also provide a viable development pathway for developing countries that are planning their energy transition toward the achievement of the sustainable development climate goals in the coming decades.

Taking China as an example, this paper attempts to answer the following three research questions: (1) to what extent will the development of green credit at different scales affect economic growth and industrial output over the next forty years; (2) what are the difference in the CO_2_ reduction effects associated with the development of green credit at different scales, and what are the green credit’s impacts on the transformation of energy structures; and (3) what mechanisms of action are responsible for the impacts of green credit on the economy and the environment, and how can the transmission effect be modelled?

Compared with econometric models, CGE models have the advantage of ex ante assessment. This is especially relevant for climate issues, which have a time horizon of decades. In many countries, decision-makers need to be provided future assessment data as a reference. In recent years, many studies have tried to construct CGE models to analyze China’s dual carbon goals without modelling the role of green finance as an essential variable [22,23,24]. Furthermore, few studies have considered the combination of economic, energy, environmental, and financial systems in the CGE model to characterize subtle interactions between the variables of the four systems.

Therefore, this paper offers three main contributions to the literature: (1) It provides a quantitative assessment of the impacts of different green credit scales on the achievement of China’s dual carbon goal, thus providing an ex ante assessment for financial policymakers. (2) This paper is the first to incorporate an innovative green credit mechanism into the previous endogenous technology CGE model [31] and endogenize the financial system. In our model, the green credit scale can affect CO_2_ emissions by directly influencing the coefficient of CO_2_ emissions and the green technology innovation curves. In sum, we integrate the energy–environmental–economic–financial (3EF) systems in the CGE model. (3) The details of our model are more realistic than those of previous models. In addition to considering green credit in the energy sector and reducing investment in traditional fossil energy, we emphasize the use of green credit in nonenergy sectors, set different carbon prices and interest rates in the equations, and consider the transformation process. 

The remainder of the paper is presented as follows: Section 2 provides the literature review and clarifies the contributions of this paper through a comparison with previous papers; Section 3 provides the main framework and equations used in this paper’s model as well as the data sources employed; Section 4 provides an in-depth discussion of the baseline and policy scenarios and provides a good rationale for the scenario-setting process employed; Section 5 presents an analysis of the simulation results to demonstrate the magnitude of the economic and environmental impacts of different green credit scales; and Section 6 summarizes the conclusions; Section 7 gives policy implications derived from the results.

## 2. Literature Review

The impacts of green finance on climate change and economic growth are important topics of global research, which are not limited to China. The Paris Agreement, signed by 178 countries in 2016, aims to limit the global temperature increase to 2 °C [32]. There is an investment requirement of USD 53 trillion by 2035 to maintain the 2 °C temperature goal. Green investments are gaining attention worldwide, with potential benefits including the development of low-carbon technologies. Closing the green financing gap could reduce unemployment and promote an increase in GDP [33]. Numerous empirical studies have examined the relationships among green finance, renewable energy, carbon dioxide, and green innovation across countries [2,34,35,36,37]. Empirical findings also support the positive effects of green finance on economic growth and environmental protection [38,39,40].

Research on green credit in developing countries is crucial due to the relatively homogeneous variety of green financial products in these countries. Green credit is an essential product and service in green finance that is mainly provided by financial institutions that offer lending services. The purpose of green credit is to integrate environmental and social responsibility into the lending and management processes of commercial banks [41]. The Equator Principles introduced environmental and social criteria into project financing for the first time, thus playing an important role in green credit development. The definition of green credit products and services differs across countries, wherein there are variations with respect to the concept of green credit in China. Countries other than China have also established policies related to green credit, such as the 2011 Bangladesh Environmental Risk Management Guidelines and the 2012 Nigerian Sustainable Banking Principles [41]. Studies conducted in other countries, such as in the UAE [42], the European region [43], and the Asian region [44], have focused on the development impact of green credit and the associated credit risks.

Theoretically, the pace of green credit development can have varying impacts on macroeconomics, the industrial structure, energy consumption, and CO_2_ emissions, thereby affecting the speed at which economies reach their carbon neutrality targets. One crucial core variable for transmission is green technology innovation. There are gaps in current green credit research.

First, most previous studies on green credit were ex post assessments that used econometrics to analyze the economic and environmental impacts and the policy transmission mechanisms associated with green credit. For example, green financial policies promote green technology innovation [45,46], which promotes efficient energy use [47], improves the energy structure, further reduces CO_2_ emissions, provides an impetus for economic growth [35,48,49], and has a great impact on the industrial structure of an economy [50]. However, the overall level of green finance development in China is low [51], the initial effect on economic growth is not significant [52], and some studies have found that the transmission pathway of green credit transmission policies is also statistically insignificant [21].

Specifically, by raising the lending threshold for polluting industries and providing convenient financing support for green industries, green credit promotes the upgrading of enterprises’ industrial structures and encourages the search for clean energy sources to replace traditional energy sources [2,47]. Thus, green credit promotes technological innovation while reducing the cost of using green products for other industries. For example, a dual-credit policy can significantly increase the number of new energy vehicles and reduce the price of supplying new energy vehicles compared to a subsidy policy [53]. The larger the green credit scales, the lower the cost of using green equipment for high-emission nonenergy sectors, and the higher the demand for use. This increase in demand can boost investment [2], thus increasing the supply of green product sectors, creating an efficient cycle, and facilitating the industrial transformation of the whole economy [17]. Overall, green credit is used to reduce the intensity of CO_2_ emissions through the effects of resource allocation and green innovation [23].

This paper uses the CGE model to study green credit policy, which is a preassessment and forward-looking method that is suitable for overall macropolicy assessment in situations where social policy experiments are difficult to conduct, particularly in carbon-neutral target policy assessment studies.

Second, few studies have used integrated CGE models to study the impacts of green credit policies. Studies have not established a CGE model to link energy, environmental, and economic systems to the respective financial system and, therefore, have not provided a systematic method of measuring the economic and environmental impacts of the development of green credit. In addition, few studies have included a detailed endogenous green credit mechanism into a CGE model. For example, the impact of a single or mixed policy of carbon tax or carbon trading on the achievement of carbon neutrality targets has been studied in recent years [54,55,56]. CGE scholars have also considered clean energy investment and green technology innovation as essential pathways to achieving carbon neutrality [57] but have not considered essential sources of technological innovation [58]. The source of green investment is limited to carbon tax recovery in a CGE model [59]. Liang et al. (2022) [31] introduced a logistic curve of endogenous technological innovation into the CGE model to better describe the pathway toward the achievement of China’s carbon neutrality goal. They empirically found that technological innovation is more effective in terms of achieving CO_2_ emissions reduction. The current study is based on Liang et al. (2022) [31], and we provide an improved CGE model compared to Liang et al. (2022).

Moreover, we incorporate the links among the energy–environmental–economic–financial (3EF) systems by introducing financial flows into the existing economic system through the addition of green credit into the financial system and identify the green credit mechanism through the CGE model so that it is possible to quantitatively assess the contributions of different green credit scales. This is one of the most critical contributions of this paper, as it allows for a quantitative assessment of the impacts of different green credit ratios on the achievement of China’s ‘double carbon’ target.

Third, research on green credit has been limited to the energy sector, with less consideration given to nonenergy sectors, such as infrastructure and construction, where green credit is more likely to be directed in China. Therefore, this paper also takes this specificity into account by distinguishing between energy and nonenergy sectors in the design of the model and adding the “adaptation costs” variable (“adaptation costs” is one of the variables added to the design of the green credit facility in this paper; the operational variables are described briefly in the next section). Additionally, the model expands on the detailed classification of the energy sector and the details of the nonenergy sector, particularly through investment and financial equations, distinguishing different green credit prices and interest rates.

Therefore, this paper develops a SAM table-based CGE model with energy and green credit details, models the mechanism of green credit, and adds it to the CGE model framework, coupling it with financial, economic, environmental, and energy systems, thereby enabling the economic and environmental impacts of green credit policy design to be assessed ex ante.

## 3. Methodological Development and Data Description

### 3.1. Design of CGE Framework

**Figure 3:**
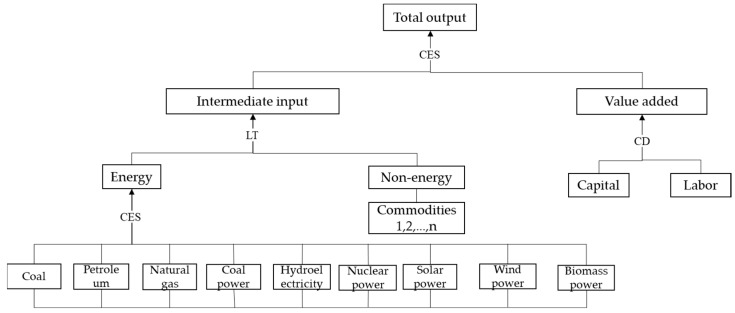
Production block of 3EF-CGE model.

Based on the model-setting method developed by Pan [60], in this study, the basic CGE blocks, including the production block, income and expenditure block, trade block, dynamic block, and closure block, were constructed. In addition to these blocks, Zhang et al. [23] and Liang et al. [31] developed expansion blocks to study the carbon neutrality problem, including the carbon tax block, carbon-trading block, energy block, and green low-carbon technology block. Based on Pan [60], Zhang et al. [23], and Liang et al. [31], this paper provides a more significant innovation for the investment block by adding a series of green credit variables to link the studied financial system with the respective economic system and establish a green credit transmission mechanism. This general framework of the CGE model used in this paper includes the production, consumption, investment, and import and export blocks. The energy block provides more details in production sectors. For example, the intermediate input energy sources were divided into coal mining, oil mining, natural gas mining, coal power, hydro power, nuclear power, solar power, and biomass power, which are assumed to be substituted among themselves in the form of the CES function (Figure 3). The energy block can be linked to the carbon block by utilizing CO_2_ emission factors. The green credit in the financial block is linked to physical investment in the real economy through investment and savings. Green credit is a critical source of green investment and can contribute to the advancement of climate-friendly technologies (green low-carbon technologies) which, in turn, affect the CO_2_ emission factor and thus the emissions reduction process. This is one of the contributions of this paper. The originality of the model presented in this paper lies in its linkage of the studied energy–environmental–economic framework (3E framework) to the respective financial system, thus forming a combination of multiple systems, the energy–environmental–economic–financial (3EF) system, and allowing for the impacts of different green credit scales on the achievement of carbon neutrality goals to be studied. The most important block included in this paper, the investment and finance block, is described below; to obtain further information regarding the other blocks and data, readers may refer to Zhang et al. [23] and Liang et al. [31].

The production block is the basic block included in the model. It shows the production behavior of enterprises. We provide the production block of the 3EF-CGE model in Figure 3. It shows the nested structure of production activities. Figure 3 shows that there is a substitutional relationship between the intermediate input and the value -added. They combine to produce the total output with the CES-productive technique. The intermediate input combines the energy and non-energy commodities with the Leontief productive technique, while the value-added combines labor and capital with the Cobb–Douglas productive technique.

### 3.2. Investment and Financial Block 

The investment block is the most essential block described in this paper and is linked to the finance block through net financial flows and the introduction of several core variables, such as the ‘green credit’ and ‘adaptation costs’. The investment equations provided herein were constructed by dividing the sectors of economic activity into energy and nonenergy sectors. This division has the advantage of more effectively differentiating between the energy and nonenergy sectors in terms of their contributions to reducing CO_2_ emissions. It also shows the difference in the costs of green credit faced by the energy and nonenergy sectors. In this paper, we provided the investment block paper with the following features: (1) we added a link between the real economy and the financial system by considering that net financial flows can be used as a source of total investment; (2) we added a link to the carbon tax system in the investment block by considering that the destination of the carbon tax collected in the carbon tax block can be used as one of the sources of total investment, thus enabling the retention of carbon revenue from the previous period in the next period, which is similar to the idea of carbon tax reinvestment [59]; (3) we allocated the total investment demand to the energy sector and nonenergy sector investments in the form of a Leontief function; (4) we introduced a series of green credit variables to the supply side of investment and considered different green credit interest rate determination methods for the energy and nonenergy sector equations; and (5) following the general dynamic mechanism, we determined that the current capital stock is equal to the previous period’s capital stock plus the current period’s investment, minus the current period’s depreciation, and the depreciation rate was determined by corresponding assumptions. Due to the unavailability of data on capital depreciation rates and sectoral instalments, different assumptions about the capital depreciation rates in the fossil energy production sector and other sectors were made, which were determined by combining available investment data with estimated industry investment trends. We assumed a capital depreciation rate of 0.05 for other sectors and a rate slightly greater than 0.05 for the fossil energy production sectors (Based on Liang et al. [31]).

**Figure 4:**
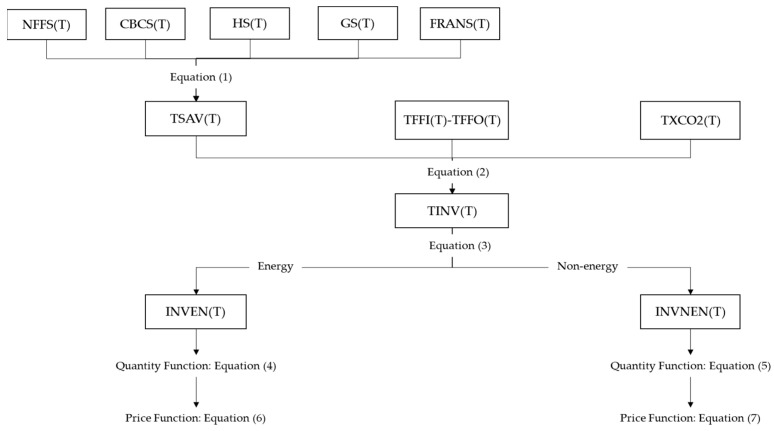
Equation’s framework and variables link in investment block.

To render the series of investment equations more comprehensible, we provide the equation-setting framework in Figure 4. 

The following are the core formulas determining the total investment and total savings.

(1) TSAV(T)=NFFS(T)+CBCS(T)+HS(T)+GS(T)+FRANS(T)

(2) TINV(T)=TSAV(T)+(TFFI(T)−TFFO(T))+TXCO2(T−1)

where *T* is the time and *TSAV(T)* is the total savings The total savings consist of nonfinancial corporate savings *NFFS(T)*, commercial bank savings *CBCS(T)*, residential savings *HS(T)*, government savings *GS(T)*, and foreign savings *FRNS(T)*. *TINV(T)* is the total investment, *TFFI(T)* is the total financial inflow, *TFFO(T)* is the total financial outflow, and *TXCO2(T-1)* is the carbon revenue of the previous period. In the original investment block, without the addition of the financial system and the carbon tax system, the total investment is equal to the total savings, but with its addition, the total investment is equal to the total savings plus the net financial inflow plus the carbon tax revenue, which constitutes the total investment demand. The total current investment minus the inventory investment is equal to the net investment, which is allocated to energy sector and nonenergy sector investment in the form of the Leontief function, and then the total energy sector investment is further allocated to more segmented energy sectors in the form of the CES substitution function. The nonenergy sector has a similar nest structure.

The total investment can be divided into two parts: the energy part and the nonenergy part.

(3) TINV(T)=INVEN(T)+INVNEN(T) 

In addition to adjusting to the investment demand, the model makes corresponding adjustments to the investment supply.

According to China’s official goal regarding carbon neutrality published on the official website of the central government of the Communist Party of China, the process of achieving carbon neutrality in China will require an adjustment in its energy structure. Specifically, the proportion of non-fossil energy consumption should reach approximately 25%, the total installed capacity of wind and solar power should reach over 1.2 billion kilowatts by 2030, and the proportion of non-fossil energy consumption should reach over 80% by 2060. Therefore, from 2020 to 2060, China’s energy investment supply will be adjusted accordingly. The investment in traditional fossil energy sources will gradually decrease and be diverted to low-carbon clean energy sectors. 

The investment block included in this paper introduces a series of green credit variables to describe the mechanism of green credit included in the CGE model, including the demand value, demand quantity, supply value, supply quantity, and the price and interest rate of green credit. The green credit rate is determined by the supply and demand of green credit, wherein the green credit rate is a two-dimensional variable that varies by sector and time.

In the corresponding equation, the green credit rate is divided into a rate for the energy sector (*GLNEN*) and a rate for the nonenergy sector (*GLNNEN*). Since there are differences in the green investment mechanisms between the energy and nonenergy sectors, we set the equations for quantity and price separately for the two sector types.

The green credit interest rate for the energy sector is determined more simply, with the quantity of green credit supplied to the energy sector (*INVENPSV1*) being equal to the quantity of demand (*INVENPSV*) through the following equation

(4) INVENPSV(PS,TH)=INVENPSV1(PS,TH)

where *PS* represents the different industry sectors, and *TH* represents the different times, starting from the 2018 base period.

The determination of the green credit interest rate in the non-energy sector has an additional variable representing the “adaptation costs”. This variable is introduced into this equation because it is considered that, in reality, the nonenergy sector uses green credit to upgrade its production equipment, for example, by gradually changing from traditional equipment to new energy equipment, which may increase or decrease the investment costs of some producers, which is partly due to the additional cost of investing in equipment to achieve a green transition. When this additional cost is positive, the cost of investing in the green transformation of equipment is higher for producers, and the price of green equipment is higher than the price of traditional equipment, which is most likely because this green equipment has not yet been popularized and its technical costs are still high. When an industry is faced with this additional cost, companies in that industry will reduce the need for “green credit” from banks, i.e., the demand for green credit will be reduced. In the early stages of the transition to a green economy, the cost of the transition will be relatively high, so the additional cost associated with the replacement of investment equipment will slowly decrease as the use of low-carbon technologies becomes more widespread.

Therefore, taking the above information into account, in our model, the nonenergy sector includes a variable representing the “adaptation cost”. *ADPC (PS, TH)* is a two-dimensional variable that varies according to sector and time, becoming smaller and decreasing in a negative power exponential form over time. The adjustment factor *f(ADPC(PS, TH))* is a function of the adaptation cost, where ORD(2018) = 1, ORD(2019) = 2, and so on, wherein ORD is an order function in GAMS, which can be used to define the base year.

(5) f(ADPC(PS,TH))∗INVNENPSV(PS,TH)=INVNENPSV1(PS,TH)

The “adaptation cost”, represented by *ADPC* (*PS, TH*), is an essential variable included in the model that affects the green credit rate. Accordingly, the quantity of green credit supplied to the nonenergy sector (*INVNENPSV1*) is equal to the quantity of its demand, with the adjustment factor *ADPC* (*PS, TH*) used to adjust the original demand (*INVNENPSV*). Thus, the adjustment factor can show the gradual expansion and development of green credit in the nonenergy sectors. The green credit rate affects the price of investment in the energy and nonenergy sectors; subsequently, the investment prices affect the prices of some variables in the production block, consumption block, foreign block, and other macroeconomic variables through the price mechanism. The equations used to show the relationship between green credit rates and investment prices are explained below for the energy and nonenergy sectors.

The investment price is a two-dimensional variable that differs by sector and time. The price of investment in the energy sector is equal to the original price (*PINVENPS0(PS, TH)*) plus the green credit rate *(GLNEN (PS, TH)* in this sector.

(6) PINVENPS(PS,TH)=PINVENPS0(PS,TH)+GLNEN(PS,TH)

However, the price of investment in the nonenergy sector is equal to the weighted average price of the original price and the price with the addition of the green credit interest rate. The weights are linked to the S-shaped technology curve for investment in the nonenergy sector, mainly because we need to reflect on the process in the long run, as the prices change through investment and technological innovations. At the beginning, when no green credit is invested in the nonenergy sector, the technology curve variable *SNCTNENPS* is equal to 0, so the price is the original price unaffected by the green credit. As the green credit is gradually increased, the green credit interest rates need to be considered. In the later stages of green credit development, the price of investment in the non-energy sector is more strongly influenced by green credit interest rates, hence the increasing weight of the green credit interest rate component.

This paper utilizes a technology curve based on life cycle theory [60,61], which depicts the entire process of technological development over time; accordingly, the investment required during this process is characterized by S-shaped development.

(7) $$\begin{array}{ll} PINVNENPS(PS, & TH)\\ & =PINVNENPS0\left(PS,\;TH\right)*\left(1-SNCTNENPS\left(PS,TH\right)\right)\\ & +\left(\left(PINVNENPS0\left(PS,TH\right)\right)+GLNNEN\left(PS,\;TH\right)\right)\\ & *\;SNCTNENPS\left(PS,TH\right) \end{array}$$

The variables presented above all have two dimensions: sector *PS* and time *TH*. *PINVENPS0(PS, TH)* and *PINVNENPS0(PS, TH)* are the investment prices of the energy and nonenergy sectors, respectively, without considering the green credit prices. *GLNEN (PS,TH)* and *GLNNEN (PS,TH)* are the green credit rates of the energy and nonenergy sectors, respectively. *SNCTNENPS(PS,TH)* is the S-shaped technology curve of the nonenergy sector.

Total green credit investment is a two-dimensional variable in the model, and its core formula can be divided into three components: *GINVPS_1_*, *GINVPS_2_*, and *GINVPS_3_*. *GINVPS_1_* is the value of investment in the nonenergy sector adjusted by the “adaptation cost” (*ADPC*) and the “S-shaped technology curve in the nonenergy sector” (*SNCTNENPS*). *GINVPS_2_* is the value of investment in the new energy sector deducted from the inventory investment. *GINVPS_3_* is the value of investment in the electricity supply sector, which has been adjusted by the S-shaped technology curve in the energy sector, and has been deducted from the inventory investment.

(8) $$\begin{array}{ll} GINVPS_{1\;}(PS,TH & )\\ & =\left(ORD{\left(TH\right)}^{-ADPC\left(PS,TH\right)}\right)*SNCTNENPS\left(PS,\;TH\right)\\ & *\;INVNENPSV\left(PS,\;TH\right)*PINVNENPS\left(PS,TH\right)) \end{array}$$

(9) $$GINVPS_{2}\;\left(PS,\;TH\right)=SINVENPS1\left(PS,\;TH\right)*\left(1-ivs\left(TH\right)\right)*TINV\left(TH\right)$$

(10) $$\begin{array}{ll} GINVPS_{3}\;(PS,\;TH & )\\ & =(SNCTENPS\left(PS,\;TH\right)*SINVENPS1\left(PS,\;TH\right)\\ & *\left(1-ivs\left(TH\right)\right)*TINV\left(TH\right)) \end{array}$$

Above, *PS* represents different industry sectors, *TH* represents the number of periods, *ivs* is the proportion of inventory investment, and *TINV* is the total investment.

Thus, the variable *ADPC(PS, TH)*, which represents the “adaptation cost” in the model, can influence the price of investment in the energy and nonenergy sectors through the green credit mechanism, thus influencing the amount of investment in the different sectors and causing substitution between energy products from the investment block to the production block, carbon block, income and expenditure block, trade block, financial block, and dynamic block.

Since the objective of this paper was to link the financial and real economic systems in the CGE model, we added an important source of net financial inflow to the total investment inflow in the investment block. The net financial inflows include the outflows and inflows of various financial investment instruments from individual agents. Our financial system extends the savings investment macro-closure of the traditional CGE model and adds the role of financial intermediation to the model. Considering that the constructed financial system needs to be linked to the extensive real economy and energy systems, we simplified the complex financial agents and financial instruments in the real world. The simplified financial system included in the model has the following assumptions: (1) the financial sector is divided into the central bank and the commercial bank; (2) the financial instruments are divided into currency, deposits, loans, deposit reserves, national bonds, financial bonds, central bank bonds, corporate bonds, equities, central bank loans, direct investment, other foreign debts, international reserve assets, and other categories; (3) the investment in each sector consists of firms’ retained earnings, equities, bonds, foreign investment, and loans; and (4) foreign investment, equities, bonds, and other financial investment instruments are assumed to be allocated in this model in fixed proportions according to the Cobb–Douglas (CD) function, and the loan amount will vary depending on the changes in exogenous interest rates.

### 3.3. Descriptions of the Basic Data 

The base data source used in the CGE model is the social accounting matrix (SAM). The SAM is a static data display of the economic system in general equilibrium that is capable of portraying the inter-relationships among economic agents, production and consumption behaviors, and factor and commodity markets. Most of the SAM data included in our model were derived from National Input–Output tables, Statement of Financial Flows, and Official Statistical Yearbooks. This paper used data from the energy SAM table presented by Liang et al. [31] as well as additional data for the financial component to achieve a match between finance and the real sector by data adjustment. The main data were obtained from the IO tables (2018) published by the National Bureau of Statistics and the funds flow tables (2018) published by the People’s Bank of China, the China Finance and Taxation Yearbook (2019), and the China Energy Statistics Yearbook (2019). All data were taken from the base year, that is, 2018.

The social accounting matrix can be conceptualized as a two-dimensional array of numbers in which each account is represented by both a row and a column. The value of each cell in the matrix displays the payment received by the account in its row from the account in its column. For each account in the SAM, the total revenue equals the total expenditure [54]. The financial SAM table constructed in this paper extends the standard SAM table with a capital account. The conversion of investment and savings is achieved through the financial system to divide the institutional account into an institutional current account and an institutional financial account. The financial SAM table contains three main components. The first part is the real economy with no savings and investment. The second part shows the changes in interinstitutional savings and their contributions to gross fixed capital formation. The third part is the financial block, which shows the flows in the financial balance sheet—the most important part of the design of variables linking the real and financial economies. Due to limitations regarding article length, the complete SAM table is included as an attachment for reference.

To achieve the research goal, the data need to be aggregated or disaggregated when developing a SAM table. Our model includes 35 sectors of production activities and 35 commodities (one-to-one correspondence). We focused on the energy sectors and nonenergy sectors with high CO_2_ emissions. Therefore, we disaggregated the electricity, heat production, and supply sectors in the IO table into Hydropower, Nuclear Power, Wind Power, Solar Power, Biomass Power, Coal Power, and the Electricity Supply, and we disaggregated the petroleum and natural gas mining sector into petroleum mining and natural gas mining with data from the China Energy Statistics Yearbook (2019). The same method was adopted to disaggregate other energy-related sectors. Data from other unrelated sectors were aggregated for simple classification. Referring to Jia and Lin [57], to make readers easier to understand this paper, we provide a list of abbreviations of the sectors in the CGE model in Table 1.

## 4. Scenario Discussion and Setting

### 4.1. Baseline

This paper explores green credit based on China’s dual carbon goals; thus, the setting of the baseline scenario for China in the coming decades is significant. The baseline model employed in this paper involves three key variables: GDP, labor, and capital. The baseline settings are fully discussed in this paper for the following reasons: first, the baseline settings are often mentioned in CGE models because they have a powerful impact on the assessment of policy effects [62], and second, the exogenous settings for the labor force population, capital, and GDP directly affect economic activity and CO_2_ emissions. Therefore, this paper compares and refers to the baseline settings used in other CGE studies on CO_2_ emissions reduction (see Table 2) before making its own assumptions.

First, regarding the baseline GDP growth rate projections, the baseline data used in the eight CGE models presented in Cao et al. [24] were taken from the China Development Research Center; these data revealed a step down from 5.5% to 2.9% from 2020 to 2050. Yuan et al. [58] compared the GDP growth rates presented by Li et al. [64] and Timilsina et al. [65], which were mostly in line, assuming an average GDP growth rate of 6.5% over the fourteenth five-year plan period, which then decreases to 5% by 2030 and 2% by 2050. This study considered the long-term planned development of China and the fact that the GDP growth rate is closely related to the country’s peak carbon emissions. Thus, we mainly used officially published data from China. In this paper, the GDP growth rate data were obtained from China’s 14th Five-Year Plan, which assumes that the GDP will double from 2020 to 2035, and that the GDP growth rate will decrease uniformly from 6% in 2019 but will always remain above 2%, with missing values supplemented by interpolation. In the baseline scenario, we also considered the impact of COVID-19 on economic growth, as there was a sharp decline in China’s GDP growth rate in 2020–2021.

Second, for the forecasting of labor force growth in the baseline period, Lin and Jia [56,63] used data on population growth from the National Population Development Plan (2016–2030); accordingly, our study also used this approach, mainly because population growth is the main driver of labor force growth. In this regard, we did not consider the impact of the quality of the labor force on human capital factors [24]. 

Third, for the prediction of the capital growth rate, the backwards projection method was adopted in this paper. Since capital growth rate data are difficult to obtain, we constructed the baseline by first using the exogenous GDP and labor force data to invert the required capital growth rate and then exogenized the obtained capital growth rate and labor growth rate to calculate the changing GDP, thus obtaining a more accurate capital growth rate under the planned GDP growth rate.

The choice of capital depreciation parameters for the model was also fully considered. Several Chinese government policies mention the need to “guide the direction of social investment, transform and upgrade traditional industries, and more strongly support the development of green and low-carbon industries such as energy conservation and environmental protection, clean production, and clean energy” to achieve carbon neutrality. This implies that future policy-driven investments in China will gradually shift from traditional energy to clean energy industries, while conventional energy equipment will be progressively eliminated. Therefore, in this study, we considered the future trend of decapitalization when setting the capital depreciation rate, i.e., investments in fossil energy will be gradually reduced and phased out faster than those in other industries. We estimate that the depreciation of the coal, petrochemical, and coal power industries will accelerate by 6.5% per year, which is higher than the 5% depreciation rate of other industries, while assuming there will be no new investment in these industries from 2028 onwards.

### 4.2. Scenario Setting

The green credit target industries are mainly clean energy industries and nonenergy industries. According to the People’s Bank of China’s Financial Institutions Loan Investment Statistics Report, at the end of 2020, the balance of China’s green loans (domestic and foreign currency) was CNY 11.95 trillion, constituting an increase of 20.3% over the beginning of the year, when the balances of loans for clean energy industries; electricity, heat, gas, and water production; supply, transportation, storage, and postal services; and green infrastructure upgrading industries were CNY 3.2 trillion, CNY 3.51 trillion, CNY 3.6 trillion, and CNY 5.76 trillion, respectively, which shows that green loans are mainly invested in these four significant industries. Therefore, according to these realistic needs, we divided all sectors in the model into green credit sectors and nongreen credit sectors. The model assumes that the investment destination of green credit is divided into three sectors: nonenergy sectors, clean energy sectors, and electricity supply sectors. The nongreen credit sectors are COL, OIL, GAS, PNC, CPR, HyP, NcP, CoP, and GPS. The green credit sector refers to the sector that can obtain green loans from banking institutions, while the nongreen sector is the sector with zero input from green credit. The green credit sectors included in the model include (1) nonenergy sectors (AGR, MTL, LIT, PAP, CMC, CST, OCI, NFR, FRR, INS, EQM, BLD, ATR, RTR, HGW, OTR, WAF, IMS, FIN, RST, EEG, and OSR); (2) clean energy sectors (WdP, SoP, and BmP); and (3) electricity supply sectors (PwS). 

If the proportion of green credit is larger, more convenient financing support for green and clean industries is provided, and for traditional high-emission industries, the interest rate increases relatively and the financing cost increases. Thus, in the long term, enterprises will be more willing to provide investments to improve the speed of technological innovation and reduce CO_2_ emissions. Eventually, the cost of products introduced by technological innovation will decrease, and the cost of green products towards green transformation for other enterprises will decrease correspondingly. Finally, this will lead to the widespread use of green products in the market, which will lead to a faster reduction in CO_2_ emissions across the whole economic system. In general, the carbon neutrality goal will be reached more quickly. In this paper, we model the transmission mechanism associated with green credit, mainly by changing the green credit scale linked to the technological progress curve, where the larger the share of green credit, the faster the technological progress. This block describes the state of climate-friendly technological progress across the whole system by introducing a logistic curve that can change the share of noncarbon energy investment and climate-friendly technology, thereby altering the CO_2_ emission coefficient and having an impact on CO_2_ emissions. Larger green credit scales can promote faster technological progress, which will accelerate the reduction in CO_2_ emissions. Thus, different green credit scales correspond to future CO_2_ emissions at different points in time and affect the timing of reaching the carbon peak and carbon neutrality goals.

This paper used the following different scenarios to reveal the impacts of policies (see Table 3). The People’s Bank of China estimates that the future green credit scale in China will be relatively easy to achieve at 60%, which is also commonly considered to be achievable by banking experts. Therefore, we used 60% as a significant policy scenario (S2) in our model, which is a typical future scenario. A pessimistic scenario (30%) and an optimistic scenario (90%) were also included in the model. The pessimistic scenario was derived from the “Commercial Bank Performance Evaluation Measures” issued by the China Banking Regulatory Commission in January 2021, which stipulates a 30% lower limit on green credit scale for banks to promote the development of green financial services in China. It was assumed that the minimum green credit scale for commercial banks will be 30% in the future, which is the most pessimistic scenario in the model. The optimistic scenario, which is different from the pessimistic and typical scenarios, assumes that 90% of future investment will be invested in the green credit sector and the remaining 10% will be invested in the nongreen credit sector. The optimistic scenario accelerates the phaseout of the conventional energy sector and the development of the clean energy sectors, and the marginal effects of different scenarios must be compared.

In this study, we assumed the green credit scale to be an exogenous variable and developed up six scenarios (see Table 3) for a policy effect comparison, including a baseline scenario, a scenario with only carbon tax and carbon-trading shocks, green credit-only scenarios (with three different scales of green credit), and a combined policy scenario. They were designed for to perform macroeconomic, industrial, and environmental effect comparisons. In scenarios S2.1–S2.3, we controlled all variables except for the green credit scale to determine the net effect of changes.

## 5. Results and Discussion

### 5.1. Macroeconomy and Industry Results

**Figure 5:**
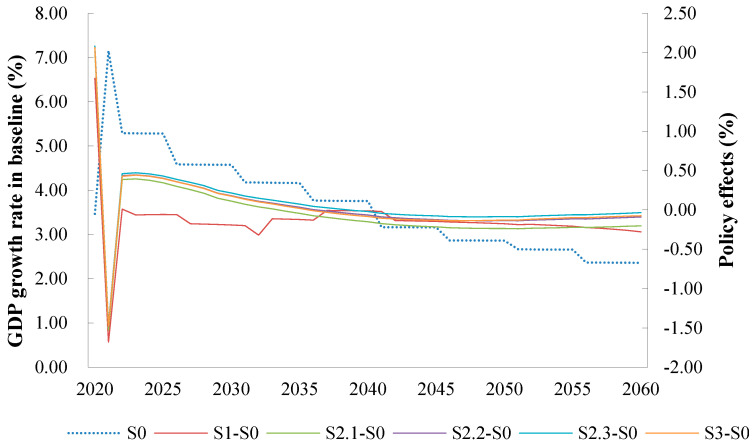
GDP growth rate projection from 2020–2060 under different scenarios in China.

From a macroeconomic perspective, green credit policies can compensate for the shortcomings of carbon tax and trading policies and further reduce GDP losses. If other conditions remain unchanged, then the larger green credit scale, the more positive the contribution to China’s GDP. Figure 5 illustrates that, without any constraints, the GDP growth rate under the natural growth path of the Chinese economy from 2020 to 2060 shows a stepwise decline with a minimum growth rate of no less than 2%, despite a slowdown in the long term. In terms of a single policy, the implementation of a carbon tax or trading policy alone would be detrimental to economic growth. In contrast, the implementation of a green credit policy simultaneously would allow for GDP losses to be offset, thus bringing the economy closer to the baseline scenario in the medium to long term.

Under the most likely combined policy scenario, S3 (the implementation of carbon tax and carbon-trading policies and 60% green credit by 2060), the projected GDP growth rate of 2.29% by 2060 is broadly in line with the S0 scenario of 2.36%, ensuring that the country’s economic growth targets will be met on schedule. Furthermore, the differences among the policy effects of the three green credit scenarios are not significant. However, as the scale of green credit investment increases, the difference between the GDP growth rate of the policy scenario and the baseline tends toward zero in the medium to long term, indicating a more balanced trade-off between economic growth and CO_2_ emissions. This is because an increase in the green credit scale, through which companies are financed, will stimulate green technology innovation and accelerate the economic upturn while minimizing economic loss due to a reduction in CO_2_ emissions.

From a sectoral output perspective (see Table 4), green credit has a negative impact on the traditional fossil energy production sector and a positive impact on the new energy production sector, as investment is gradually shifted from sectors with high CO_2_ emissions to sectors with low CO_2_ emissions, which is more contributory to the development of the new energy sector. As the green credit scale increases, the overall negative impact gradually decreases, with a positive output from most sectors, except for the traditional energy sector.Due to the effects of technological progress, the larger the green credit scale, the faster green investment stimulates green technology progress and the more significantly it boosts GDP growth.

**Figure 6:**
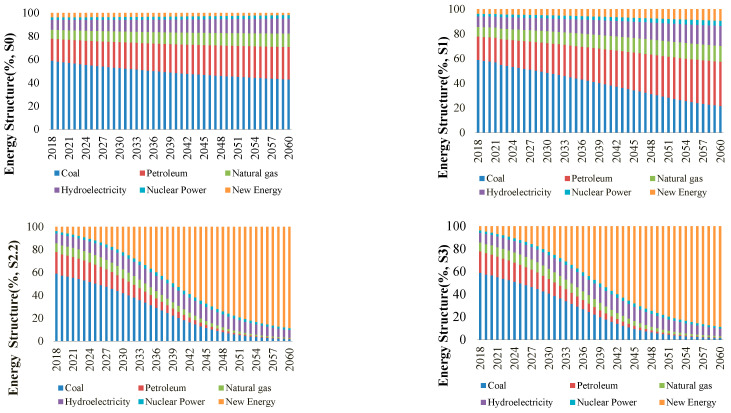
Energy structure transformation based on four scenarios in China.

This also corresponds to the changes in the GDP shown in Figure 6. In S2.2 (Table 4), with only 60% green credit, the production levels in the wind, solar, and biomass power generation sectors rise by 322.82%, 234.09%, and 461.43%, respectively, by 2060 relative to the baseline scenario, while production in the coal power generation sector falls by 61.32%. The decrease in the output of traditional fossil energy is lower than the increase in the output of the new energy sector. The main reason for this is that the traditional fossil energy sector has a large capital stock, and if it only has ordinary depreciation rates, then it is not destocking fast enough. On the other hand, the new energy sector has a small capital stock at the beginning of the investment period, and the capital increases quickly and thus its output will increase rapidly. Overall, the rapid development of new energy sources offsets the GDP lost by phasing out traditional energy, resulting in a benefit to the macroeconomic output as a whole.

The results of the combined scenario (S3) differ to a lesser degree from those of the scenario with the implementation of green credit policies only (S2.1–S2.3), and to a greater degree from those of the carbon tax and carbon-trading scenario (S1), especially in terms of the magnitude of the increase in production in the new energy sector and the contraction in production from traditional energy sectors. The comparison of the scenario results shows that the implementation of carbon tax and carbon-trading policies alone plays a minor role in achieving a long-term energy transition, while the simultaneous consideration of green credit policies and green innovation promotion will play a more significant role in the long term.

### 5.2. Heterogeneity Analysis of the Change in the Energy Structure

From 2020 to 2026, under the baseline scenario without carbon goal constraints and energy policy controls, China’s energy structure is predicted to change slowly, with the share of coal use gradually declining but still dominating energy use, i.e., it is estimated that by 2060, coal use will still account for 42.88% of energy use, and oil and natural gas use will reach 27.95% and 11.54%, respectively. No significant change in the total share of traditional fossil energy is predicted, and this remains at over 80%. Over the next 40 years, hydropower, nuclear power, and new energy sectors with low CO_2_ emissions will not be well developed. This shows that if the initiative is not taken with respect to carrying out economic transformation, the energy demand will not automatically shift to new energy sectors. The reduced share of coal will be replaced by other traditional fossil and clean energy sources with technical and cost advantages. The new energy industry cannot compete with other energy sources due to its weak technology and capital accumulation. The industrial development of new energy is limited, and the energy structure will not fundamentally change.

The implementation of the green credit policy will promote the green transformation of China’s energy structure. The energy structures S2.2 and S3 are similar. From 2020 to 2060, China’s energy structure will change into an inverted S shape, with coal, oil, and natural gas production declining, while new energy production will rise in an S shape. With the participation of green credit policies, the Chinese economy will achieve a significantly faster energy transition, with the proportion of traditional fossil energy sources decreasing at an accelerated rate and the share of coal decreasing from 59% in 2020 to 1.56% in 2060. Over the next 40 years, the proportion of new energy will show an S-shaped increase, replacing traditional fossil energy well.

**Figure 7:**
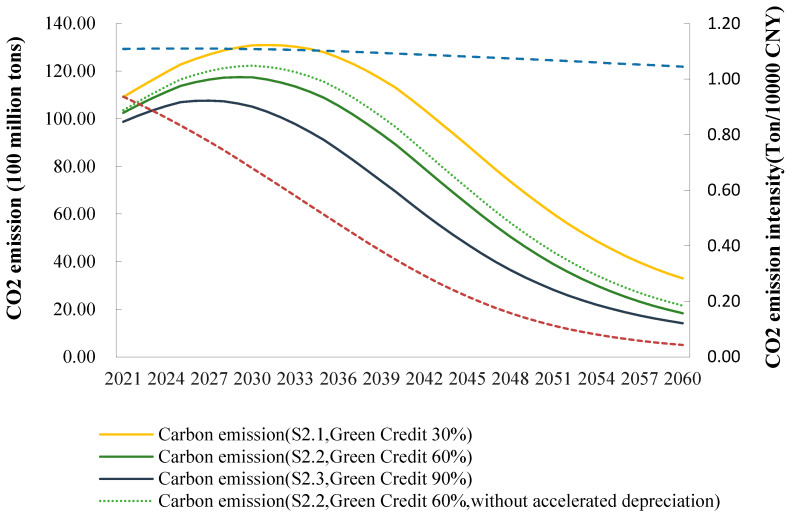
Change in CO_2_ emissions and intensity, 2020–2060, China.

The share of new energy is predicted to grow from 3.75% in 2020 to 88.59% in 2060. The energy shares of hydropower and nuclear power will not change significantly, with the main changes being a slight increase at the beginning of the time period and then a decrease, which is mainly because green credit is not invested in the nuclear and hydrosectors in our model setup. Our empirical study shows that the speed of the energy transition in S1 is not as fast as in S2.1, S2.2, and S2.3 (Figure 7). In addition, one of the carbon neutrality targets in China is to attain a non-fossil energy share of more than 80% by 2060. This target cannot be achieved in S0 and S1 but can be achieved in S2 and S3, suggesting that the contribution of green credit policies to the achievement of long-term energy transition targets is not negligible. 

### 5.3. The Change in CO_2_ Emissions

It is clear from the baseline scenario that if the Chinese government does not adopt green policies to control CO_2_ emissions in the long term, energy use will not transition from conventional to clean energy sources, and CO_2_ emissions will accumulate to an unsustainable level. Furthermore, the literature [24,31] confirms that if we only employ both carbon tax and carbon-trading policies without other policies, effective carbon reduction will not be achieved in the medium to long term, nor will the 2060 carbon neutrality goal be achieved. Therefore, green credit policies should be designed to guide the flow of funds in different industries and improve the level of green technology innovation achieved by enterprises to achieve the carbon neutrality goal on schedule. The use of different green credit scales in the future will result in different levels of CO_2_ emissions and carbon intensity, hence encouraging the achievement of the carbon peak and carbon neutrality goals.

The larger the green credit scale, the less time it takes to peak, and the lower the peak value. Figure 7 shows the trends in CO_2_ emissions and intensity from 2020 to 2060 for different green credit sizes. Figure 7 shows that, under different green credit sizes, CO_2_ emissions peak in approximately 2030 and then slowly decline, although the rates of peak and decline differ. In S2.3, the time to peak (2027) is four years earlier than in S2.1 (2031), with carbon peaking at 10.76 and 13.10 billion tons, respectively. In S2.2, the peak year is 2029, with CO_2_ emissions peaking at 11.75 billion tons, which is close to China’s 2030 carbon peak goal.

The larger the green credit scale, the more beneficial it is to achieve the carbon neutrality goal in China. In S2.1, S2.2, and S2.3, CO_2_ emissions are reduced to 3.3, 1.83, and 1.42 billion tons by 2060, respectively. S2, which only includes green credit, shows a significant slope decrease in the CO_2_ intensity compared to the baseline scenario S0 from 0.93 tons/CNY 10000 in 2021 to 0.04 tons/CNY 10000 in 2060. The above results suggest that the larger the green credit scale, the more it scale promotes green and low-carbon technological progress, thereby accelerating the overall macroeconomic carbon reduction process.

In addition, the carbon reduction effect is weakened if the accelerated depreciation condition is not incorporated. In S2.2, new investment in the traditional fossil energy sectors has to stop before achieving carbon peaks goal, and the depreciation of capital stock will accelerate before new energy sources can successfully replace traditional fossil energy. Otherwise, the energy transition will not be achieved spontaneously. Therefore, the carbon reduction results for S2.1–S2.3 shown in Figure 7 also incorporate a decapitalization factor, i.e., an accelerated depreciation rate for capital stock in the coal, petroleum and petrochemical, and coal power sectors of 6.5% annually. This is higher than the 5% depreciation rate of other sectors. At the same time, no new investment will be made in these sectors from 2028 onwards. If the capital stock in the traditional energy and fossil sectors is not depreciated at an accelerated rate or still receives new investment to continue expansion, the model cannot converge to an equilibrium status. This is mainly because the coal, coal power, and petrochemical industries maintain their technological and cost advantages, and new energy sources cannot replace traditional fossil energy and rely solely on market forces. The carbon reduction effect will be significantly reduced.

**Figure 8:**
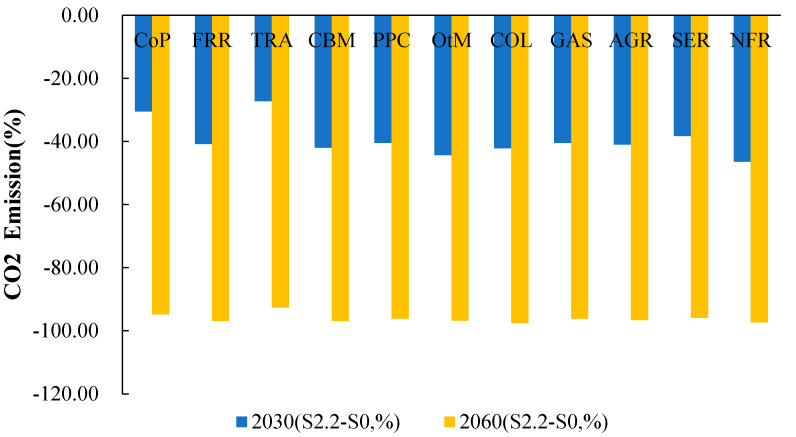
CO_2_ emission percentage change of 11 major sectors in 2030 and 2060, China. Note: TRA includes ATR, RTR, HGW and OTR; CBM includes CST and BLD; PPC includes OIL and PNC; OtM includes all other manufacturing sectors except above sectors; SER includes all services sectors.

In the long term, green credit effectively and sustainably reduces CO_2_ emissions throughout the Chinese economy. To visualize the percentage change in CO_2_ emissions from the major sectors, we aggregated the 35 sectors into 11 main sectors in the model. Figure 8 shows the percentage change in CO_2_ emissions for the 11 industries at two key time points, 2030 and 2060, for the S2.2 (60% green credit) scenario. Compared to the baseline scenario without any policy shocks, the green credit policy scenarios effectively reduce CO_2_ emissions in various sectors, especially in sectors that produce high quantities of CO_2_ emissions, such as the coal, steel, and nonmetallic manufacturing sectors (including cement). For example, in S2.2, the 60% green credit policy scenario, compared to the baseline scenario S0, CO_2_ emissions in 2060 are reduced by 94.80%, 96.93%, and 97.32% in the coal-, steel-, and nonmetallic manufacturing sectors, respectively, while other sectors are predicted to have similarly significant carbon reduction effects of over 90% by 2060. Clearly, the sustainability of the role played by green credit is non-negligible. That is, it has a long-term carbon reduction effect, which gradually becomes stronger over time. In S2.2, the carbon reduction effect in 2060 is more than double that in 2030, especially for the infrastructure sector, which is a nonenergy sector. The long-term carbon reduction effect is significant, e.g., −27.23% and −41.92% for the transport and construction sectors in 2030, respectively, and −92.62% and −96.89% in 2060, respectively.

### 5.4. The Expected Carbon Goal

The implementation of a green credit policy will facilitate the achievement of phased goals, final goals, and China’s long-term carbon neutrality goal over the next 40 years. In China’s officially published planning documents for the energy transition and dual carbon goals, four key indicators have been targeted: CO_2_ emissions per unit of GDP (carbon intensity), energy consumption per unit of GDP, the share of non-fossil energy consumption, and the quantity of CO_2_ emissions, with the key time points being 2025, 2030, and 2060. The target values relate to three stages: the short (2025), medium (2030), and long terms (2060). We list the simulation results for these four key targets in Table 5.

The simulation results obtained in this study show that by implementing a green credit policy only, whether the green credit scale is 30%, 60%, or 90%, all goals can be achieved in the short term (2025), except for the goal regarding the CO_2_ emissions per unit of GDP (carbon intensity). However, in the medium to long term (2030–2060), green credit policies will play a critical role in achieving the carbon goal. For example, in S2.2 (Table 5), under the scenario of 60% green credit scale, the carbon intensity in 2030 is predicted to be 76.45% lower than in 2005, exceeding the target value of “65%” by 11.45%. The share of non-fossil energy consumption is predicted to be 46.75%, exceeding the target value of “25%” by 21.75%. It is worth noting that although the green credit scale does not have a significant impact on the achievement of the target values, it still shows a specific pattern in the simulation results: as the green credit scale increases, the reduction in emissions is more significant, and the carbon target value can be reached more quickly, i.e., the time taken to reach the target is reduced. This means that the larger the green credit scale, the more quickly the carbon neutrality goals can be achieved.

Additionally, the green credit scale has a significant impact on the CO_2_ emissions goal. For the peak carbon goal, the green credit scale scenarios of 30%, 60%, and 90% are predicted to result in 13.08, 11.74, and 10.52 billion tons of CO_2_ emissions by 2030, with the projected peak carbon years occurring in 2031, 2029, and 2027, respectively (see Table 5). The 60% green credit scenario is closer to China’s “2030 carbon peak” goal. This suggests that a large increase in the green credit scale will help shorten the time taken to reach the carbon peak goal.

For the carbon neutrality goal, the green credit scale is closely related to the time taken to achieve the goal. Carbon neutrality means that net CO_2_ emissions reach zero, i.e., the quantities of emitted and absorbed CO_2_ offset each other. We expected a final outcome of zero CO_2_ emissions in our model. However, in this paper, the CO_2_ emissions remained at nonzero values, because our model does not include policies such as carbon capture, forest carbon sinks, and ocean carbon sinks. Therefore, a final CO_2_ emissions value of zero was not predicted for 2060. However, these results are still reasonable. In S2.2 and S2.3, the remaining 1.833 and 1.423 billion tons of CO_2_ emissions could be absorbed through the implementation of carbon sink policies, thus allowing China’s carbon neutrality goal to be achieved.

### 5.5. Model Validation with the Green Credit Interest Rates

**Figure 9:**
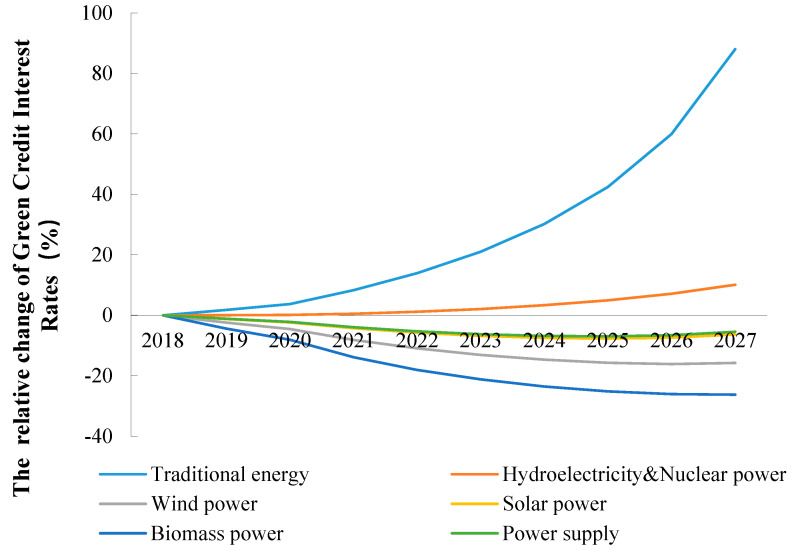
The relative change in green credit interest rates for energy sectors compared to benchmark, 2018–2027, China.

This section shows the endogenous variable green credit interest rates derived for scenario S2.2 (60%) to provide evidence for the model’s validation. It is assumed that from 2028 to 2060, there is no green credit offered to the traditional energy sector, so Figure 9 only provides the relative change in the green credit interest rate for the energy sector for 2018–2027. The benchmark interest rate is 1. In 2019, the green credit interest rate for conventional energy increased by 1.76% over the benchmark in S2.2, with a green credit size of 60%. It is then projected to increase exponentially to 88.02% by 2027, which will be driven by the combined policy that China uses to pursue the attainment of peak carbon status by 2030. The green credit interest rates for the hydroelectricity and nuclear power sector are also predicted to increase slightly (by 10.12%) over the benchmark by 2027. The green credit interest rates for the solar power, wind power, and biomass sectors all predicted to decrease, with the most significant reduction occurring for biomass power, followed by wind power and solar power. By 2027, the green credit interest rates for the biomass, wind, and solar power industries are predicted to decrease by 26.26%, 15.76%, and 6.35%, respectively, compared to the benchmark. We compared the green credit rates for the relevant energy industries in the Chinese market in 2019, 2020, and 2021 and found that the green credit rates determined by our model did not differ significantly from them, which laterally confirms the reliability of the current model.

## 6. Conclusions

Green credit could accelerate China’s achievement of its carbon neutrality goal. Green credit has an effect on emissions reduction, economic growth, and energy transition. 

We showed that the scaling up of green credit can accelerate the pace of green technological advancements, leading to a faster reduction in CO_2_ emissions. From an empirical point of view, in contrast to [2,20], we considered the role of green finance and carbon-pricing policies in achieving the dual carbon goals. Unlike other empirical studies of green technology innovations [16,62], our model quantifies a green credit mechanism in the integrated CGE model and establishes a 3EF integrated system, which can be used to evaluate the effect of the green credit scale policy in advance. We found that technological innovations can offset a loss of Gross Domestic Product (GDP) during the energy transition process. Green credit can bring stable economic growth to China while also helping the country attain its carbon neutrality goal.

Apart from the mutually beneficial outcomes of emissions reduction and economic growth, green credit can also encourage a crucial shift in the energy mix, with increased consumption of clean energy and a decrease in brown energy consumption. Under the most likely combined policy scenario, S3 (implementing the carbon tax and carbon-trading policies and 60% green credit in 2060), the projected GDP growth rate of 2.29% by 2060 is broadly in line with the S0 scenario of 2.36%, thus ensuring that the country’s economic growth targets will be met on schedule. In this scenario, the share of fossil energy is predicted to drop from 80% to 20% by 2060 with the rapid development of new energy sources, which may account for up to 90% of China’s total energy production. The economy will enter a sustainable and high-quality development phase.

Furthermore, we have provided evidence that can be used to determine the green credit scale for the policy bank. When determining the best green credit scale for achieving the dual carbon goals, the marginal decreasing effects cannot be ignored. Under a cost–benefit perspective, a green credit size of 60% is the most likely proportion that can be realistically achieved in China, and in this scenario, the dual carbon goals can be achieved. In addition to the determination of the green credit scale, decision-makers should also consider destocking the traditional energy sector to render the green credit policy effective.

## 7. Policy Implications

In the coming decades, the scaling up of green credit in commercial banks is a Chinese policy whose implementation is designed to achieve the country’s dual carbon goals. 

The execution of carbon tax and carbon-trading policies, or a combination of the two, will help to achieve the carbon peak goal but will not lead to carbon neutralization. In the long term, China needs technological progress as a solid backing to reach its long-term carbon neutrality goal, but technological progress requires strong financial support. To promote the development of green finance, government instruments such as subsidies and incentives can be utilized. However, due to the huge financial gap within the market, the reliance on solely government instruments is an inadequate measure with which to satisfy the overall demand for green credit. Consequently, it is imperative to use market instruments and expand the green finance scale. During the initial phase of green finance development in China, green investment with the objective of stimulating green innovation in industries was primarily facilitated through the provision of green credit by commercial banks.

However, financial risks should be a concern. During the implementation of green credit scale policies, economies are exposed to risks in addition to positive impacts. For example, there is a risk of strong contraction in the traditional fossil energy sectors, which could easily lead to the removal of funding and generate a risk of bad debt for commercial banks. The transition risk will be transmitted to non-energy industries with high-carbon emissions simultaneously, such as the cement, chemical, and transportation industries. It will then be transmitted to the asset portfolios of financial institutions, and financial system risks may occur. To assess the potential impact of carbon neutrality on the asset quality of financial institutions, microlevel data may be employed to track changes in customer credit ratings and other relevant metrics. This analysis enables the identification of the risk exposure and asset loss of financial institutions, thus providing regulators with a more accurate basis for developing stress test scenarios to guide financial institutions in the management of climate-related risks. One of shortcomings in this study is that we only include one financial risks indicator in the model. This is what can be improved in future studies.

There is a vast gap in capital in the green financial market in China. A robust green credit market should be established. Furthermore, there is scope for the incorporation of various green financing instruments, such as green bonds, green funds, and green insurance, into the financial market. Capital markets should be used to drive the advancement of green technology innovation, thereby facilitating energy transition, climate change mitigation, and sustainable economic development.

In sum, to achieve carbon neutrality, China should adopt a multistage approach. Initially, policy-based carbon reduction measures should be implemented, followed by the adoption of technical carbon reduction measures during the intermediate phase. In the final stage, negative carbon technology approaches should be employed. It is important to note that each of these stages necessitates significant investments in green credit to eventually attain net zero emissions and successfully accomplish China’s carbon neutrality goal.

## Figures and Tables

**Table 1:** Sector Classification.

Sector Number	Abbreviations	Non-Energy Production Sectors
1	AGR	Agriculture, hunting, forestry, and fishing
5	MTL	Metal and nonmetal
6	LIT	Light industry
7	PAP	Paper and paper products
10	CMC	Chemical
11	BLD	Building materials
12	OCI	Other chemical industry
13	NFR	Nonferrous
14	FRR	Ferrous
15	INS	Ships and related installations
16	EQM	Other equipment manufacturing
25	CST	Construction
26	ATR	Air transport
27	RTR	Railway and urban rail
28	HGW	Highway
29	OTR	Other transportation, warehousing, postal services
30	WAF	Wholesale and retail trade and accommodation and food service
31	IMS	Information service
32	FIN	Finance
33	RST	Real estate and leasing
34	EEG	Ecological protection and environmental governance
35	OSR	Other public services
		Fossil Energy Production Sectors
2	COL	Coal mining
3	OIL	Petroleum mining
4	GAS	Natural gas mining
8	PNC	Petrochemicals and nuclear
9	CPR	Coal processing
22	CoP	Coal power
24	GPS	Gas production and supply industry
		New Energy Production Sectors
19	WdP	Wind power
20	SoP	Solar power
21	BmP	Biomass power
23	PwS	Power supply
		Other Energy Production Sectors
17	HyP	Hydroelectricity
18	NcP	Nuclear power

**Table 2:** Comparison of CGE model baseline settings.

Reference	Model	Research	The Basic Assumption of Baseline
Cao et al. [24]	8 Chinese CGE models developed for model comparison exercise	A multimodel comparison of China’s carbon tax policy under carbon neutrality goal	GDP growth rate and population growth projection data, all from the China Development Research Center, wherein GDP growth is assumed to be 5.5% for 2020–2025, 4.5% for 2025–2030, 4.0% for 2030–2035, 3.4% for 2035–2045, and 2.9% for 2045–2050.
Yuan et al. [58]	Chinese CGE model with an endogenous energy-based technological change	Impact of carbon-pricing policies and non-fossil energy incentives on the achievement of carbon-peaking and carbon neutrality goals	GDP growth is assumed to be 6.5% in 2020–2025, decreasing to 5% by 2030 and gradually decreasing to 2% by 2050.
Lin and Jia [56,63]	A dynamic recursive CGE model focusing on the impact of carbon tax on energy, environment, and economy; Chinese CGE model developed to analyze the impact of national carbon-trading scheme	Assessment of the economic, environmental, and energy impacts of different tax rates and carbon tax regimes in China; assessment of the economic, energy, and environmental impacts of China’s national carbon-trading market	The labor force is exogenous and is set based on the National Population Development Plan (2016–2030); capital depreciation is determined by the current capital stock and investment, which is endogenous except during the first period; investment is also endogenous.

**Table 3:** Green credit scenario setting.

Scenario	Explanation
S0(Baseline)	Natural growth scenario without any policy shocks
S1(Carbon tax and carbon trading)	Only carbon tax and carbon trading, no green credit
S2.1 (Green credit 30%)	Only green credit, growing linearly from 7% green credit in 2018 to 30% in 2060
S2.2 (Green credit 60%)	Only green credit, growing linearly from 7% green credit in 2018 to 60% in 2060
S2.3 (Green credit 90%)	Only green credit, growing linearly from 7% green credit in 2018 to 90% in 2060
S3 (Combined policy)	Scenario S1 + S2.2

**Table 4:** Percentage change of industrial output and prices in 2060, China.

Sector Number	Non-Energy Production Sectors	2060 Industrial Output (Quantity Percentage Change)				
		S1	S2.1	S2.2	S2.3	S3
1	AGR	1.50	2.06	2.21	1.81	2.22
5	MTL	−4.35	−10.25	−4.51	0.69	−3.31
6	LIT	−7.15	−3.21	−0.07	2.65	0.05
7	PAP	−10.21	−6.75	−1.82	2.60	−1.64
10	CMC	−9.08	−9.74	−4.65	0.15	−4.11
11	BLD	−25.01	−10.79	−4.01	2.25	−2.92
12	OCI	−16.95	−9.89	−3.73	1.91	−3.19
13	NFR	−6.18	−4.26	1.51	6.55	1.70
14	FRR	−19.39	−11.00	−4.39	1.70	−3.65
15	INS	−11.70	−7.28	−2.12	2.49	−1.99
16	EQM	−6.56	−1.42	2.72	6.32	2.88
25	CST	−18.43	−8.20	−2.90	1.87	−1.94
26	ATR	−21.58	−6.44	−4.28	−2.19	−1.06
27	RTR	−11.49	−8.81	−3.17	2.14	−3.14
28	HGW	−15.10	−8.97	−3.81	1.05	−3.36
29	OTR	−14.09	−10.21	−5.17	−0.34	−4.60
30	WAF	−9.69	−4.63	−0.65	3.00	−0.42
31	IMS	−8.56	−3.17	0.43	3.64	0.54
32	FIN	−10.77	−6.38	−2.02	2.17	−1.78
33	RST	−8.85	0.21	3.68	6.86	3.73
34	EEG	−8.93	−5.19	−0.98	2.89	−1.09
35	OSR	−9.32	−5.37	−1.77	1.57	−1.81
	Fossil Energy Production Sectors	S1	S2.1	S2.2	S2.3	S3
2	COL	−57.75	−68.30	−73.59	−78.00	−77.12
3	OIL	7.68	−90.94	−91.74	−92.45	−91.78
4	GAS	−7.32	−88.99	−89.98	−90.85	−35.11
8	PNC	−13.86	−52.38	−60.59	−67.32	−56.91
9	CPR	−77.78	−74.84	−79.23	−82.87	−82.52
22	CoP	−30.00	−62.99	−61.32	−61.22	−62.65
24	GPS	21.59	−43.16	−45.31	−46.23	−41.75
	New Energy Production Sectors	S1	S2.1	S2.2	S2.3	S3
19	WdP	37.96	331.84	322.82	302.18	327.12
20	SoP	37.92	241.03	234.09	218.08	237.46
21	BmP	37.74	473.52	461.43	433.64	467.29
23	PwS	−15.27	13.64	66.54	134.88	64.38
	Other Energy Production Sectors	S1	S2.1	S2.2	S2.3	S3
17	HyP	43.40	136.86	129.26	116.23	131.07
18	NcP	39.64	133.26	127.86	116.59	129.89

**Table 5:** Comparisons among different scenarios and expected goals.

**Year**	**Change in CO_2_ Emissions per Unit of GDP (%)**				
	**Goal**	**S0**	**S2.1 (30%)**	**S2.2 (60%)**	**S2.3 (90%)**
2025	18% lower than 2020	−1.49	−11.54	−14.83	−15.76
2030	65% lower than 2005	−61.58	−74.00	−76.45	−79.00
2060	significantly lower than that in 2020	−7.28	−92.16	−95.61	−96.48
**Year**	**Change in energy consumption per unit of GDP (%)**				
	**Goal**	**S0**	**S2.1 (30%)**	**S2.2 (60%)**	**S2.3 (90%)**
2025	13.5% lower than 2020	−1.40	−10.23	−11.08	−11.51
2030	a sharp decline	−1.14	−19.22	−20.56	−21.26
2060	a sharp decline	−1.10	−13.12	−17.81	−20.97
**Year**	**Proportion of non-fossil energy consumption (%)**				
	**Goal**	**S0**	**S2.1 (30%)**	**S2.2 (60%)**	**S2.3 (90%)**
2025	20%	21.00	32.26	32.86	33.15
2030	25%	22.00	45.64	46.75	47.28
2060	85%	25.00	97.66	97.94	98.05
**Year**	**CO_2_ emission (100 million tons)**				
	**Goal**	**S0**	**S2.1 (30%)**	**S2.2 (60%)**	**S2.3 (90%)**
2030	Carbon peak	185.38	130.81	117.39	105.23
2060	Carbon neutrality	444.74	32.98	18.33	14.23

## Data Availability

The data used to support the findings of this study are available from the corresponding author upon request.

## Supplementary Materials

The following supporting information can be downloaded at: https://www.mdpi.com/article/10.3390/ijerph20054508/s1, Table S1. SAM table in the model. Table S2. SAM table in the model (Continuing table).
