# Supplementary material for: Can Green Credit Policies Accelerate the Realization of the Dual Carbon Goal in China? Examination Based on an Endogenous Financial CGE Model
Source: Int J Environ Res Public Health. 2023 Mar 3;20(5):4508. doi: 10.3390/ijerph20054508 (PMC10002116; doi:10.3390/ijerph20054508)
Supplement: Supplementary file 1 [file ijerph-20-04508-s001.zip › ijerph-2227769-supplementary.pdf]

Table S1. SAM table in the model.

		Production activities	Products	Factors of production		Current accounts of institutions						Capital accounts of institutions		Financial accounts of institutions					
				Labour	Capital	Non-financial companies	Central Bank	Commercial Banks	Households	Government	The rest of world	Fixed capital formation	Changes in inventories	Non-financial companies	Central Bank	Commercial Banks	Households	Government	The rest of world
Production activities			Products supply																
Products		Intermediate input							Households consumption	Government consumption	Export	Investment demand	Changes in inventories						
Factors of production	Labour	Labour remuneration																	
	Capital	Capital income				Capital income													
Current accounts of institutions	Non-financial companies									Capital transfers									
	Central Bank							Reserve for deposits											
	Commercial Banks																		
	Households			Labour remuneration	Capital income	Corporate transfer			Inter-resident transfers of income and expenditure	Government transfers to residents									
	Government	Production taxes	Goods and			Income		Income taxes	Income taxes										
	The rest of world		Import							Government transfers abroad									
Capital accounts of institutions	Fixed capital formation											Changes in inventories		Actual investment	Actual investment	Actual investment	Actual investment	Actual investment	Actual investment
	Changes in inventories																		
Financial accounts of institutions	Non-financial companies					Total corporate capital													
	Central Bank																		
	Commercial Banks							Total capital of commercial banks											
	Households								Total resident capital										
	Government									Total government capital									
	The rest of world										Total foreign capital								
Financial flow accounts	Currency													Currency			Currency	Currency	Currency
	Deposits													Deposits			Deposits	Deposits	Deposits
	Loans															Loans			Loans
	Deposit reserves														Deposit reserves				Deposit reserves
	National bonds													National bonds		National bonds	National bonds		National bonds
	Financial bonds													Financial bonds		Financial bonds	Financial bonds	Financial bonds	Financial bonds
	Central bank bonds														Central bank loans				Central bank bonds
	Corporate bonds															Corporate bonds	Corporate	Corporate bonds	Corporate bonds
	Equities													Equities		Equities	Equities	Equities	Equities
	Central bank loans														Central bank bonds				
	Direct investment													Direct investment					Direct
	Other foreign debts													Other foreign debts		Other foreign debts			Other foreign debts
	International reserve assets														International reserve assets				
	Other categories													Other categories		Other categories	Other categories	Other categories	Other categories
Total	Total expenditure	Total production	Total supply	Elemental compensation		Institutional expenses						Total saving		Changes in institutional assets					

Table S2. SAM table in the model (Continuing table).

		Financial flow accounts														Total
		Currency	Deposits	Loans	Deposit reserves	National bonds	Financial bonds	Central bank bonds	Corporate bonds	Equities	Central bank loans	Direct investment	Other foreign debts	International reserve assets	Other categories	Total income
Production activities																Total output
Products																Total demand
Factors of production	Labour															Primary income
	Capital															
Current accounts of institutions	Non-financial companies															Institutional income
	Central Bank															
	Commercial Banks															
	Households															
	Government															
	The rest of world															
Capital accounts of institutions	Fixed capital formation															Total investment
	Changes in inventories															
Fiancial accounts of institutions	Non-financial companies			Loans					Corporate bonds	Equities		Direct investment	Other foreign debts		Other categories	Changes in institutional liabilities
	Central Bank	Currency			Deposit reserves										Other categories	
	Commercial Banks		Deposits				Financial bonds	Central bank bonds		Equities	Central bank loans		Other foreign debts		Other categories	
	Households			Loans											Other categories	
	Government			Loans		National bonds							Other foreign debts		Other categories	
	The rest of world		Deposits	Loans		National bonds	Financial bonds	Central bank bonds	Corporate bonds	Equities		Direct investment	Other foreign debts	International reserve assets	Other categories	
Financial flow accounts	Currency															Financial inflows
	Deposits															
	Loans															
	Deposit reserves															
	National bonds															
	Financial bonds															
	Central bank bonds															
	Corporate bonds															
	Equities															
	Central bank loans															
	Direct investment															
	Other foreign debts															
	International reserve assets															
	Other categories															
Total	Total expenditure	Financial outflows														
